# Dulce Digital-Me: protocol for a randomized controlled trial of an adaptive mHealth intervention for underserved Hispanics with diabetes

**DOI:** 10.1186/s13063-021-05899-x

**Published:** 2022-01-28

**Authors:** Athena Philis-Tsimikas, Addie L. Fortmann, Job G. Godino, James Schultz, Scott C. Roesch, Todd P. Gilmer, Emilia Farcas, Haley Sandoval, Kimberly L. Savin, Taylor Clark, Mariya Chichmarenko, Jennifer A. Jones, Linda C. Gallo

**Affiliations:** 1 Scripps Whittier Diabetes Institute, Scripps Health, San Diego, USA; 2 Laura Rodriguez Research Institute, Family Health Centers of San Diego, San Diego, USA; 3 Herbert Wertheim School of Public Health and Human Longevity Science, University of California San Diego, San Diego, USA; 4 Neighborhood Healthcare, San Diego, USA; 5 San Diego State University/University of California San Diego Joint Doctoral Program in Clinical Psychology, San Diego, USA; 6 Department of Psychology, San Diego State University, San Diego, USA; 7 Qualcomm Institute, University of California, San Diego, USA

**Keywords:** Digital, Diabetes, Hispanic, Latino, HbA1c, Health behavior, Cost-effectiveness

## Abstract

**Background:**

By 2034, the number of US individuals with diabetes is predicted to increase from 23.7 to 44.1 million, and annual diabetes-related spending is expected to grow from $113 to $336 billion. Up to 55% of US Hispanics born in the year 2000 are expected to develop diabetes during their lifetime. Poor healthcare access and cultural barriers prevent optimal care, adherence, and clinical benefit, placing Hispanics at disproportionate risk for costly diabetes complications. Mobile technology is increasingly prevalent in all populations and can circumvent such barriers. Our group developed Dulce Digital, an educational text messaging program that improved glycemic control relative to usual care. Dulce Digital-Me (DD-Me) has been tailored to a participant’s individual needs with a greater focus on health behavior change.

**Methods:**

This is a three-arm, parallel group, randomized trial with equal allocation ratio enrolling Hispanic adults with low income and poorly managed type 2 diabetes (*N* = 414) from a San Diego County Federally Qualified Health Center. Participants are randomized to receive Dulce Digital, Dulce Digital-Me-Automated, or Dulce Digital-Me-Telephonic. The DD-Me groups include Dulce Digital components plus personalized goal-setting and feedback delivered via algorithm-driven automated text messaging (DD-Me-Automated) or by the care team health coach (DD-Me-Telephonic) over a 12-month follow-up period. The study will examine the comparative effectiveness of the three groups in improving diabetes clinical control [HbA1c, primary outcome; low-density lipoprotein cholesterol (LDL-C), and systolic blood pressure (SBP)] and patient-provider communication and patient adherence (i.e., medication, self-management tasks) over 12 months and will examine cost-effectiveness of the three interventions.

**Discussion:**

Our comparative evaluation of three mHealth approaches will elucidate how technology can be integrated most effectively and efficiently within primary care-based chronic care model approaches to reduce diabetes disparities in Hispanics and will assess two modes of personalized messaging delivery (i.e., automated messaging vs. telephonic by health coach) to inform cost and acceptability.

**Trial registration:**

NCT03130699-All items from the WHO Trial Registration data set are available in https://clinicaltrials.gov/ct2/show/study/NCT03130699.

**Supplementary Information:**

The online version contains supplementary material available at 10.1186/s13063-021-05899-x.

## Background and rationale

The prevalence of diabetes is growing and is projected to increase by nearly 50% to 629 million worldwide by the year 2045 [1–6]. In the USA, care for people with diagnosed diabetes accounts for one in four health care dollars with an estimated total economic cost of $327 billion in 2017—26% higher than the previous estimate in 2012 [7]. Diabetes imposes substantial burden on society and significant health disparities are experienced by individuals of low-income and racial/ethnic minority groups [5, 8–10]. Prevalence of diagnosed diabetes is substantially higher among people of Hispanic ethnicity (12.5%) compared to non-Hispanic whites (7.5%) [11–14]. In addition to greater prevalence, Hispanics also exhibit poorer clinical management and outcomes once diagnosed with type 2 diabetes (T2D) compared to non-Hispanic whites [15–17].

Maintaining good risk factor control reduces the risk of micro- and macrovascular diabetes complications [18–21], and lowers short- and long-term medical costs [22, 23]. Nonetheless, many individuals with diabetes do not achieve recommended targets [24]. In the 2011–2014 National Health and Nutrition Examination Survey (NHANES), approximately half (47.9%) of adults aged 20 years and older with diabetes had HbA1c < 7%, and only 20.7% met all targets for HbA1c, lipids, and blood pressure (BP) [15, 25]. Risk factor control was poorer in Hispanics compared with non-Hispanic whites [15, 25], a finding consistent with substantial prior research [15]. Unfortunately, many primary care physicians—especially those who care for medically under-resourced populations—are challenged to meet these goals, as clinical endpoints can be affected by social determinants and behavioral factors unrelated to the treatment provided [26].

Diabetes self-management education and support (DSME/S) is a cornerstone of effective care that leads to improved adherence, clinical, quality of life, and cost outcomes [24]. Despite the demonstrated effectiveness of DSME/S for improving T2D outcomes and reducing disparities, many at-risk individuals are unable to access it due to practical barriers (e.g., work, transportation, caregiving). To improve patient and practice performance outcomes, providers need alternative methods to efficiently and effectively extend the reach of DSME/S.

Mobile health (mHealth) technology is rapidly evolving and has the capacity to positively impact outcomes in diabetes [27–29]—even among at-risk groups. In 2021, Hispanics and non-Hispanic whites reported very high and roughly equivalent rates of cellphone ownership (97–100%) [30]. The Pew Research Center has also reported Hispanics (73%) to be more likely than non-Hispanic whites (58%) to use a mobile phone to seek health information [31]. While investigations of mHealth for diabetes are limited in Hispanics [32], recent, small studies provide preliminary evidence that simple, text message interventions can achieve clinically significant improvements in glycemic control in this population [33–35]. The widespread adoption of mobile phones—even among older adults and individuals from low-income and racial/ethnic minority groups—and the expressed desire of many to leverage technology to manage their personal health highlights the potential for mHealth technology to circumvent the practical barriers inherent to traditional (e.g., face-to-face) DSME/S among Hispanics [36]. Nonetheless, larger and longer, methodologically rigorous randomized controlled trials (RCTs) are needed to confirm the value and acceptability of mHealth interventions in this at-risk population.

This 5-year, RCT was developed on the backbone of three programs with proven efficacy in integrating glucose management and digital technologies in underserved communities—Project Dulce, Dulce Digital, and CYCORE (*CY*berinfrastructure to support *CO*mparative effectiveness *RE*search) [37]. *Project Dulce* is a diabetes care and education program that addresses the specific needs of culturally diverse populations. The Project Dulce, evidence-based curriculum is recognized by the American Diabetes Association and Medicare and has been shown to improve clinical, behavioral, and cost outcome in underserved, predominantly Hispanic adults with T2D [38–45]. *Dulce Digital* is a text message-based version of the Project Dulce curriculum that includes “static” educational and motivational messaging and prompts to take medication and check blood glucose. Similar to Project Dulce, Dulce Digital has been shown to improve glycemic control in the low-income Hispanic population in Southern California [34]. *CYCORE* is a secure, scalable, and extensible platform that is device agnostic with the ability to support adaptive or “dynamic” mHealth interventions [37]. CYCORE can accommodate both inbound data capture from the glucose and medication monitors, and support the logic behind the text message-based ecological momentary assessment (EMA) and interactive communication (i.e., personalized feedback and goal-setting) with study participants. The Dulce Digital-Me (DD-Me) intervention that is under evaluation in the current trial represents a marriage of Dulce Digital and CYCORE. By capitalizing on the dynamic properties of CYCORE, the original Dulce Digital texting program has been enhanced to incorporate “adaptive” or dynamic components (e.g., personalized feedback) [34, 46, 47], which may hold advantages over static/one-size-fits-all approaches [29, 48–52]. This randomized comparative effectiveness study will not only evaluate the additive value of personalized assessment, goal-setting, and feedback to a text message-based diabetes program, but will also examine two different methods of delivering these adaptive components: automated text messaging (Dulce Digital-Me-Automated) versus telephonic delivery by a medical assistant (MA) Health Coach (Dulce Digital -Me-Telephonic). Importantly, we will also capture patient and provider needs and perceptions, costs, and other practical implications for integrating these technologies into underserved populations and real-world health care practices.

## Study aims

The primary aim is to compare the effectiveness of Dulce Digital, Dulce Digital-Me-Automated, and Dulce Digital-Me-Telephonic in improving diabetes clinical management [HbA1c—primary outcome; low-density lipoprotein cholesterol (LDL-C), and systolic blood pressure (SBP)] over 12 months. We hypothesized that the Dulce Digital-Me-Automated and Dulce Digital-Me-Telephonic groups would both show greater improvements in clinical management over 12 months relative to the Dulce Digital group. We did not have an a priori prediction regarding superiority of the Dulce Digital-Me-Automated versus Dulce Digital-Me-Telephonic groups.

The following are secondary and exploratory aims:

Secondary

- To compare the effectiveness of Dulce Digital, Dulce Digital-Me-Automated, and Dulce Digital-Me-Telephonic groups in improving patient adherence and patient-provider communication over 12 months. We hypothesized that the Dulce Digital-Me-Automated versus Dulce Digital-Me-Telephonic groups would both show greater improvements in patient adherence and patient-provider communication over 12 months relative to the Dulce Digital group. We did not have an a priori prediction regarding superiority of the Dulce Digital-Me-Automated versus Dulce Digital-Me-Telephonic groups.
- To examine the cost-effectiveness of the Dulce Digital, Dulce Digital-Me-Automated, and Dulce Digital-Me-Telephonic groups.

Exploratory

- To examine whether effectiveness, cost and/or acceptability differ significantly between Dulce Digital-Me adaptive feedback *methods* [i.e., automated text messaging versus health coach telephonic delivered].

## Trial design

**Fig. 1:**
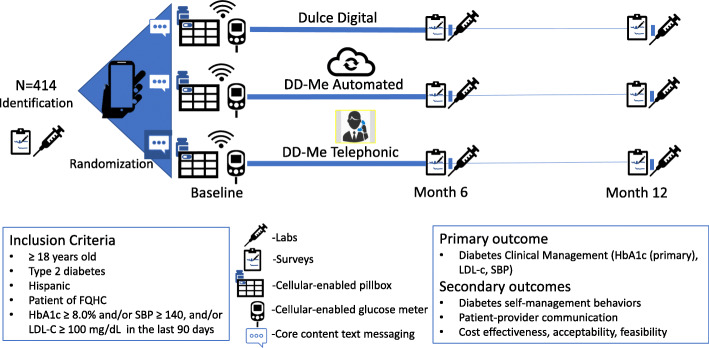
DD-Me trial design. After identification and randomization, all participants are provided a cellular-enabled pill box, glucose monitor and (if needed) mobile phone. All received Core Content text messages and were asked to answer ecological momentary assessments (EMA) over 6 months. Pill box, glucose levels, and EMA item responses were used in the Dulce Digital-Me-Automated and Dulce Digital-Me-Telephonic groups to formulate adaptive messaging during the 6-month intervention period. Follow-up labs and surveys were conducted at months 6 and 12. DD-Me-Dulce Digital-Me, FQHC-Federally Qualified Health Center, HbA1c-glycosylated hemoglobin, LDL-c low-density lipoprotein cholesterol, SBP-systolic blood pressure

This is a three-arm, randomized (with equal allocation), parallel group comparative effectiveness trial with *N* = 414 participants with assessors only blinded to allocation (Fig. 1). The protocol was developed in accordance with Good Clinical Practice, SPIRIT, and CONSORT 2013 guidelines.

## Methods: participants, interventions, and outcomes

### Study setting

The study setting and participant enrollment site is Neighborhood Healthcare, a Federally Qualified Health Center (FQHC) that encompasses 11 health centers located throughout Northern San Diego and Southern Riverside counties. Neighborhood Healthcare is a private, nonprofit community health system and a designated Patient-Centered Medical Home, which serves as a safety net for the community. Neighborhood Healthcare serves high proportions of un- and underinsured patients and medically underserved ethnic/racial minority groups (i.e., > 60% of patients, of whom 80% are Hispanic). Neighborhood Healthcare uses a single electronic health record (EHR) system across all sites.

### Partners

This study is the product of a Southern California academic-healthcare-community partnership between Scripps Health, a large, nonprofit health system; community partner, Neighborhood Healthcare; and academic partners, San Diego State University and University of California, San Diego.

#### Stakeholder engagement-community advisory board

Consistent with community-engaged research principles [53–55], patients with T2D and members of the Neighborhood Healthcare clinical care team (i.e., MAs, nurses, primary care providers) and leadership were involved in study planning and have been engaged as Community Advisory Board (CAB) members to optimize implementation and sustainability, and facilitate dissemination efforts. Further, the development of the Dulce Digital-Me approach has been guided by input from providers (e.g., requested a greater focus on health behavior change) and patients from the original Dulce Digital trial (e.g., desired a more “personalized” and adaptive intervention tailored to their needs and progress). The CAB was convened quarterly during study startup to refine and finalize the approach and has continued to receive annual communication for the remainder of the study to guide implementation. The CAB will also eventually contribute to efforts to disseminate finding and sustain and scale the intervention beyond the research period.

### Eligibility criteria

The target population for this study includes Hispanic/Latino adults (ages 18 years or older) who are registered patients of Neighborhood Healthcare, with a diagnosis of T2D, and at least one of the following within 90 days of enrollment: HbA1c ≥ 8.0% *and/or* SBP ≥ 140, *and/or* LDL-C ≥ 100 mg/dL. Exclusion criteria are as follows: severe illness precluding regular clinic visits (e.g., serious malignancy; end stage liver or kidney disease; severe cognitive impairment); pregnant or lactating; type 1 or gestational diabetes; lack of minimal literacy needed to participate in the text intervention; severe auditory or visual problems; primary language other than Spanish or English; not willing to carry a mobile phone; plans to relocate.

### Sample size

This study will enroll *N =* 414 men and women allocated equally to groups. RMASS2 [56, 57] was used to estimate the sample size needed to detect statistically significant differences between any of the 3 groups: (a) Dulce Digital, (b) Dulce Digital-Me-Automated (automated text message delivery), and (c) Dulce Digital-Me-Telephonic (telephonic MA health coach delivery). RMASS2 is designed to accommodate longitudinal data with attrition when a comparison between groups is of primary interest. Our power analysis for HbA1c, as the primary clinical outcome, is presented here for exemplary purposes. A clinically meaningful change of 0.5% with a 1.3% standard deviation was used. To transform this estimate into an effect size (*d* = .33), standard deviations (SD) from prior studies in the same [34] and similar populations [45] were used. To determine the minimum sample size necessary, additional assumptions were made: (1) an alpha level of .05 and a power level of .80; (2) a missing data rate of 15% at each time-point or a 30% missing data rate overall, and (3) a stationary autoregressive structure (lag 1) for the variance-covariance matrix of the repeated measures, using an autocorrelation value of .45. Given these assumptions and the estimated effect size from above, 414 participants are needed at baseline (i.e., *n* = 138 in each of the 3 groups). Although power analyses used HbA1c as the primary outcome, *N* = 414 at baseline will achieve power > .80 to detect a small-to-medium effect size for all physiological (HbA1c, LDL-C, SBP) and all patient-reported outcomes, given a missing data rate of 30% over the course of the study.

### Recruitment, screening, and enrollment

Automated, EHR-derived patient identification reports were developed in collaboration with the FQHC EHR analysts. Patient identification reports are delivered weekly and include all patients meeting criteria according to demographic, diagnostic, and clinical indicators noted above. Potentially eligible individuals are contacted by telephone by trained, bilingual research staff who provide a brief overview of the study and screen for additional eligibility criteria (e.g., minimal literacy, no plans to relocate) and interest. A recruitment script is followed and screening database is maintained consistent with CONSORT guidelines [58, 59]. Those who are eligible and interested are invited to an initial visit, at which the study is fully described, written informed consent is obtained, baseline assessments are performed, and participants are randomized.

#### Informed consent

The research assistant reviews a paper copy of the informed consent document with the patient in their preferred language. During the consenting process, research assistants monitor the participant’s comprehension and halt the consenting process if a participant demonstrates difficulty understanding content and meaning of the study or informed consent document. If the participant has temporary difficulties reading (e.g., did not bring eyeglasses), the consent form is read aloud word for word in the presence of a witness (e.g., family member). Once ample opportunity to ask questions about the study is provided, participants are asked to provide written informed consent. For participants who decline participation, a reason for refusal is collected.

#### Brief educational session

After randomization, all participants view a 20-min educational presentation that gives an overview of diabetes management basics, including topics of physical activity, nutrition, blood glucose monitoring, medication adherence, and emotional well-being. The presentation also reinforces study processes and intervention components that participants can expect (e.g., study devices, staff support and outreach, and who to call for any study-related questions). After the slide presentation, research assistants provide individualized device training, with opportunities for participants to practice, receive feedback, and independently demonstrate device competence. Following the training, the participants receive paper copies of the slides that have device use instructions. See Appendix A for Education Session topics.

### Intervention groups

**Fig. 2:**
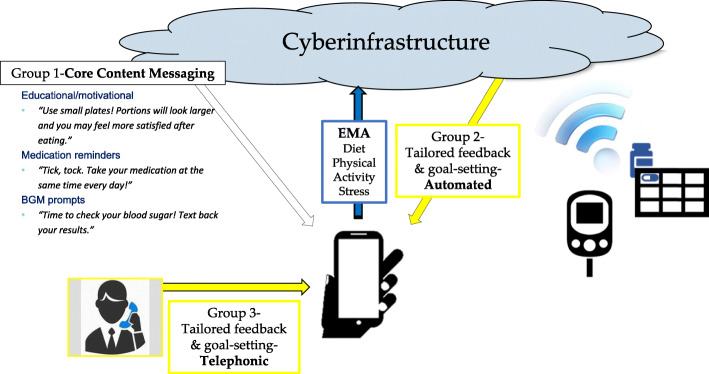
Conceptual overview of DD-Me trial intervention arms. After randomization, participants in the three groups receive all core components noted in the figure (i.e., core content messaging, cellular-enabled pill box, cellular-enabled glucose meter). Participants are assigned to receive either static, core content text messages alone (Group 1 Dulce Digital), or core content messaging *plus* adaptive automated text messages (Group 2 DD-Me-Automated) or adaptive, MA health coach telephonic delivered messaging (Group 3 DD-Me-Telephonic) based on information collected from the cellular-enabled pill box and glucose monitor and responses collected from the ecological momentary assessments. EMA ecological momentary assessments

At the conclusion of the Initial Visit, participants are randomly assigned to one of three groups: Dulce Digital (Group 1), Dulce Digital-Me-Automated (Group 2), or Dulce Digital-Me-Telephonic (Group 3) (Fig. 2).

All interventions are implemented between baseline and month 6 (Fig. 1). Participants in all groups receive a cellular-enabled Telcare blood glucose monitor (BioTelemetry, Inc, Malvern, PA) and enough strips for (up to) twice-daily blood glucose testing for the duration of the 12-month study, and a cellular-enabled pill box (WisePill Technologies, Western Cape, South Africa) to manage oral medication(s) and monitor adherence. Participants who lack a personal cell phone with texting capabilities are provided with one. Those who own a personal cell phone but lack an adequate text messaging plan are provided with financial compensation to cover plan enhancement. All participants continue to receive evidence-based diabetes care at the clinic for the duration of the study. Medication use or other interventions are left to the physician’s discretion and are tracked for study purposes. To facilitate patient-provider communication and accommodate physician stakeholder requests, primary care physicians are provided with a summary of blood glucose values and behavioral data from the CYCORE system prior to clinic visits (Appendix B). Table 1 provides an overview of all Intervention Components by groups. In all cases, intervention content was delivered in the participant’s preferred language, Spanish or English. Content was translated by certified translators to Spanish before implementation.

**Table 1:** Overview of intervention components

Intervention component	Dulce Digital (Group 1)	DD-Me (Groups 2 and 3)
Core Content Messaging	**X**	**X**
BG Monitoring with triage	**X**	**X**
EMA of health behaviors and emotional well-being	**X**	**X**
Medication Adherence Monitoring	**X**	**X**
Adaptive Feedback & Goal-Setting		**X** ^**a**^

Notes. *BG* blood glucose, *DD-Me* Dulce Digital-Me, *EMA* ecological momentary assessment

^a^Delivered by automated text messaging delivery in Group 2 and via telephonic MA health coach delivery in Group 3

#### Group 1, Dulce Digital

Participants in this group receive the original Dulce Digital, culturally and health literacy-appropriate text messages between baseline and 6 months (“Core Content”). Over this same period, participants are encouraged to regularly check glucose using the cellular-enabled Telcare monitor (“Remote Glucose Monitoring”), manage their oral medication(s) using the cellular-enabled Wisepill box (“Remote Medication Adherence Monitoring”), and respond to ecological momentary assessment (“EMA”) questions about health behaviors and well-being via text message. Each Dulce Digital component is described in greater detail below.

##### Core content messaging

In Group 1, Dulce Digital participants receive a total of 254 core content text messages covering five diabetes domains that align with the Association of Diabetes Care and Education Specialists (ADCES) 7 Self-Care Behaviors [60]: (1) Medication Adherence; (2) Clinical Indicators; (3) Dietary Behaviors; (4) Physical Activity; (5) Stress/emotional distress. Two to three messages per day are sent at study start at timeslots pre-selected by the participant, with frequency tapering over 6 months. As a “static” intervention, all Dulce Digital participants receive the same messages in terms of dosage, content, and order. Table 2 details the outgoing text message frequency, by message type and intervention group and Appendix C provides examples of text message content.

**Table 2:** Outgoing text message frequency, by message type and intervention group

	Weeks 1–5^a^	Weeks 6–10^a^	Weeks 11–15^a^	Weeks 16–20^a^	Weeks 21–24^a^	Total
Core content messages	13	13	11	9	6	254
EMA items	3	3	3	3	3	72
EMA feedback messages^b,c^	3	3	3	3	3	72
BG feedback messages^b,d^	1	1	1	1	1	24
Medication adherence feedback messages^b^	1	1	1	1	1	24
Totals						
Group 1/Dulce Digital	16	16	14	12	9	326
Group 2/DD-Me automated	21	21	19	17	14	446
Group 3/DD-Me-Telephonic	16	16	14	12	9	326

Notes. *BG* blood glucose, *DD-Me* Dulce Digital-Me, *EMA* ecological momentary assessment

^a^Frequencies reflect number of items or messages delivered each week

^b^Received only by Group 2/DD-Me, automated

^c^EMA feedback message was only sent if participant responded to a particular EMA item

^d^The weekly BG feedback messages in this table are distinct from any real-time/safety messages that are sent in response to critical BG values (as described in “Remote blood glucose monitoring” section).

The core content messaging is informed by several empirically based behavioral and learning theories, including Operant Conditioning [61, 62], Social Cognitive Theory [63], Social Ecological Model [64, 65], Theory of Planned Behavior [66, 67], and Transtheoretical Model/Stages of Change [68]. Each core content message was also mapped onto Abraham and Michie’s taxonomy, which classifies 93 distinct behavior change techniques into 16 domains [69–71].

##### Remote blood glucose monitoring

A personalized text message is sent in week 2 of the intervention to each participant, indicating the number of times they should plan to check their blood glucose weekly based on their baseline HbA1c (see Table 3): “Make a goal to check your blood sugar X times per week (about X times each day).”

**Table 3:** Recommended blood glucose monitoring frequency, by baseline HbA1c

	Baseline HbA1c			
	< 7%	7–8.4%	8.5–10%	> 10%
Number of blood glucose checks per week	6	9	12	14

All blood glucose values taken by the participant are automatically transmitted in real time from the Telcare monitor to CYCORE via cellular connectivity. As a safety protocol, participants receive an automated/real-time text message encouraging provider follow-up if they meet any of the following criteria: 1 value < 57 mg/dL; 3 values 57–70 mg/dL in last 14 days; or 3 values > 250 mg/dL in last 14 days [e.g., “Your blood sugars have been running high. Your medication may need a change. Call your medical provider to discuss ways to bring your values to goal (80-180)”]*.* As an added safety measure, study staff receives automated email alerts from the CYCORE platform when any of the aforementioned criteria are met *or* if no values transmitted for 1 week. In each of these scenarios, a study staff member calls the participant to briefly assess the possible reasons for hypo/hyperglycemia (or lack of monitoring) and encourages follow-up with a provider as needed. Importantly, the as-needed telephone outreach described here for the Dulce Digital group is narrowly focused on safety, engagement, and technology/device troubleshooting; it does not include tailored goal-setting and feedback strategies as received by the Dulce Digital-Me groups.

##### Remote medication adherence monitoring

All participants are instructed to use the WisePill box to manage their anti-hyperglycemic agent(s). If participants were not prescribed anti-hyperglycemic agents, they were instructed to use the WisePill box for their anti-hypertensive or cholesterol medications. WisePill is a portable medication dispenser with an embedded cellular data connection that does not require the use of a smartphone or expensive cellular data plan. Each time the dispenser is opened, WisePill transmits a time-stamped event record to the CYCORE system. If CYCORE does not receive a WisePill interaction for 14 days, an alert email is sent to prompt study staff to reach out to the participant to encourage device usage and/or troubleshoot technical difficulties as needed. Similar to blood glucose monitoring and triage, these calls do not include goal-setting or feedback techniques that are used in the DD-Me groups.

##### Ecological momentary assessment of diet, physical activity, and emotional well-being

All participants receive three EMA items via text message each week; one item/week assesses dietary behaviors (i.e., fruit, vegetable, whole grain intake; meal composition and portions; reduction of saturated fat, sugar, sodium), one item/week assesses physical activity (i.e., aerobic exercise; flexibility and strength activities; reduction of sedentary time), and one item/week assesses emotional well-being (i.e., current/recent distress, stress, and motivation levels; recent use of positive coping strategies). To limit burden, all EMA items are designed to only require a single character response from the participant to reflect the number of days (0–7) in the past week they have engaged in the target health behavior; whether or not they have engaged in the health behavior (Y/N) in the last 24 h; or their level of emotional well-being or distress on a 10-point Likert Scale (0-10). Each time an item is answered, the participant’s response is transmitted in real time as a time-stamped event record to the CYCORE system. While EMA items are completed by all participants for evaluation purposes, by design, participants in the Dulce Digital group do not received feedback on their EMA responses. If CYCORE does not receive an EMA item response from a given participant for 14 days, an alert email is sent to prompt study staff to reach out to the participant to encourage responding and/or to troubleshoot texting difficulties as needed.

#### Group 2, Dulce Digital-Me, automated text messaging feedback

To accommodate patient and provider feedback and consistent with research demonstrating the value of adaptive interventions, Dulce Digital-Me was designed to include all Dulce Digital components described above *plus* adaptive behavioral feedback and goal-setting. To further enhance personalization, participants in this group are also afforded the opportunity to select which one of the five core content domains they wish to receive first; aside from ordering, there were no changes to content or dosage from the Dulce Digital group.

##### Adaptive feedback and goal-setting

The adaptive behavioral feedback and goal-setting component is facilitated by the CYCORE system and is informed by participants’ real-time (1) responses to diet, exercise, and well-being EMA items sent via text message, (2) wirelessly transmitted blood glucose values; and (3) wirelessly transmitted medication adherence data from the Wisepill box. Operant Conditioning theory purports that actions that are followed by reinforcement (e.g., praise, tangible reward) will be strengthened and more likely to occur again in the future [61, 62]. With mHealth, behavioral progress can be monitored and reinforced in real time via algorithm-driven messaging. As participants attain smaller self-management goals (related to medication adherence, diet, exercise, healthy coping), receive encouraging feedback, and improve their health, they are expected to experience a reduction in perceived barriers and improved self-efficacy.

Thus, each time a Group 2/Dulce Digital-Me-Automated participant responds to an EMA item via text message, CYCORE transmits an automated feedback/goal-setting message in real time. This message is specifically tailored to reinforce each participant’s unique, self-reported progress on that behavioral domain. A priori behavioral targets were defined to categorize responses into “optimal,” “near optimal,” “sub-optimal,” and “needs improvement” self-management (see Table 4). If/then logic and response message sets were designed specifically to motivate maintenance of optimal/near-optimal progress (Optimal example: “That’s fantastic! Keep up the good work. Physical activity is a key to better health!”) and to increase engagement in areas where participants’ responses to EMA items suggest sub-optimal progress/a need for improvement (Sub-Optimal example: “That’s a good start! For best health, try to be active 5-7 days per week. What additional exercise can you add?”).

**Table 4:** Behavioral and glucose targets used to determine goal-setting/feedback provided to DD-Me-Automated participants

	Optimal	Near optimal	Sub-optimal	Needs improvement
EMA item responses				
Number of days/week	5–7 days/week	3–4 days/week	1–2 days/week	0 days/week
Dichotomous (Yes/No)	Yes			No
BG control	> 75% of values 80–180 mg/dL	50–75% of values 80–180 mg/dL	25–49% of values 80–180 mg/dL	< 25% of values 80–180 mg/dL
Medication adherence	7 days/week	5–6 days/week	3–4 days/week	< 3 days/week

Notes. *BG* blood glucose, *DD-Me* Dulce Digital-Me, *EMA* ecological momentary assessment

Dulce Digital-Me-Automated participants also receive goal-setting/feedback messages on their wirelessly transmitted blood glucose and medication adherence data. To reduce message burden, glucose control and medication adherence feedback are transmitted by the CYCORE system on a weekly basis and summarizes progress in these areas over the past 7 days. Similar to the EMA feedback, a priori targets were set for glucose control and medication adherence (see Table 4), and if/then logic and response sets were developed to motivate maintenance of optimal/near-optimal glucose control and medication adherence (Optimal examples: “Most of your sugars were in the 80-180 mg/dL target range this week. This isn’t always easy to do; be proud of yourself!” and “Excellent! You took your medication every day this week! You are taking an important step to protect your health!”), as well as to increase engagement when glucose control or medication adherence falls in the sub-optimal/needs improvement ranges (Needs improvement examples: “Very few of your blood sugars were in the 80–180 mg/dL target range this week. Can you think of changes you can make to improve this?” and “You only took your pills on less than 3 days this week. It is important to take them every day. Call your medical provider if you have questions.”).

#### Group 3, Dulce Digital-Me-Telephonic MA Health Coach feedback

In Group 3/ Dulce Digital-Me-Telephonic participants, the adaptive feedback and goal-setting is delivered weekly, via telephone, by a MA Health Coach as opposed to the CYCORE algorithm-driven automated messaging. For rapport-building, at the conclusion of the Initial visit, Group 3 participants are introduced to the DD-Me MA Health Coach, or (when the MA is not available) are provided a handout with her picture and description of role. In order to minimize differences in feedback provided other than delivery modality, the MA Health Coach receives a weekly report to guide their feedback calls (see Appendix D). The MA Health Coach Report integrates data from the same sources (EMA, glucose meter, Wisepill box) and applies the same a priori behavioral and BG targets as described above for the automated messaging group. The MA Health Coach completes intervention logs detailing the duration and content of this outreach.

### Technical platform development

**Fig. 3:**
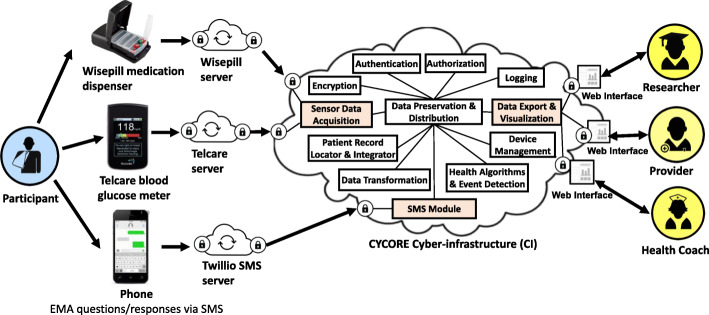
CYCORE Interactive System. Technical overview of CYCORE interactive system: (1) Data acquisition from Wisepill medication dispenser, Telcare blood glucose meter, and phone EMA; (2) CYCORE Cyberinfrastructure services for managing data, devices, and users; and (3) data visualization for each user role: researcher, healthcare provider, and Health Coach.2

CYCORE Cyberinfrastructure supports the collection, storage, visualization, analysis, and interpretation of physiologic and behavioral data (Fig. 3). The CYCORE system integrates information from three sources: (1) Wisepill box for medication adherence data, (2) Telcare meter for blood glucose data, and (3) cell phone for receiving intervention messages (core content information, medication feedback, blood glucose feedback, and EMA items) and for replying to EMA items. The CYCORE Cyberinfrastructure achieves coherent system integration out of a variety of distributed components and manages the lifecycle of all resources, while addressing crosscutting concerns such as security, policy management, and logging. The CYCORE user interface mandates role-based access and provides tailored views for each role (e.g., researcher, provider, and MA Health Coach).

CYCORE is a secure, scalable and extensible platform with the ability to support adaptive or “dynamic” mHealth interventions. The SMS (i.e., short message service, or text) module handles the logic of the text messaging interaction with participants. A database stores intervention messages, feedback algorithms or “rules,” and participant responses to messages. Messages are encoded by date/time, sequence number, and behavioral pattern. The SMS Module uses this information to determine the appropriate message to send to each participant at the requested time. When the participant’s reply is received, the system matches it with the question that is pending response. Furthermore, the content of the reply is compared against the rules to determine what (if any) additional message should be sent. CYCORE also contains tools that enable monitoring of the intervention. EMA questions have an expiration time, and a response is considered invalid if the participant replies after the message is expired. The system flags erroneous or unexpected responses from participants and sends email alerts to the research team, potentially indicating an individual having difficulties with the system.

### Study devices

#### Blood glucose monitor

All blood glucose values taken by the participant using the study-assigned devices are wirelessly transmitted in real time from the Telcare Verizon blood glucose meter (BioTelemetry, Inc, Malvern, PA) to CYCORE, and made available to the study team.

#### Wireless medication adherence device

WisePill Dispenser Int 3G (WisePill Technologies, Western Cape, South Africa) is a portable medication dispenser with an embedded cellular data connection that does not require the use of a smartphone or expensive cellular data plan. Each time the dispenser is opened, WisePill transmits a time-stamped event record to the CYCORE system and will trigger outgoing algorithm-based text message in the same manner as for EMA data.

#### Cellphone

All intervention content and feedback is delivered through a cell phone. Participants not owning a cell phone received an option of two different types of cell phones, a BLU Jenny (Doral, Florida) or an LG Expression with a QWERTY keyboard (Englewood Cliffs, New Jersey). Wireless service was provided by U.S. Mobile with unlimited text and voice plans for the duration of the intervention. Data plans were not provided to any study participants.

### Intervention monitoring, adherence, and withdrawals

#### One-week technology check call and device retraining

One week after intervention start, a research assistant contacts the participant to ensure that all devices are working properly and that the participant is able to properly work the devices, and to answer any questions that participants may have. If a participant is experiencing any difficulty, a research assistant will either provide the participant with additional training over the phone or arrange another in-person one-on-one training with the participant. If the issue is due to a faulty device, participants will be provided with a replacement.

#### Participant withdrawals

Participants who request to no longer receive the intervention are closed out at that time and are referred to as voluntary withdrawals. Follow-up outcome assessments will be completed at months 6 and 12 for participants who are reachable and agreeable. If the participant dies or requests to be withdrawn from the study with no further contact, these participants are referred to as administrative withdrawals and no follow-up assessments are attempted. Institutional Review Board approval was received to audit the EHR for laboratory and anthropometric values collected as part of standard of care that can be used in place of (missing) research follow-up assessments.

#### Concomitant interventions

Dulce Digital-Me is conducted in the context of the usual and customary outpatient health care encounters care provided by Neighborhood Healthcare. The trial provides adjunct services, and all participants are expected to continue to receive care as usual, in accordance with their primary care plans. There is no restriction placed on concomitant interventions that may be obtained.

### Interventionist training and support

#### Medical assistant health coach training and supervision

The MA health coach completes a 3-day “Fundamentals of Diabetes Management” course delivered by Scripps Registered Nurse/Certified Diabetes Care Education Specialist (RN/CDCES). Pre-post measures assess knowledge and skills gained. Training days 4–5 combine didactics with experiential exercises to educate the MA on the basics of operant conditioning and behavioral shaping and combines didactics with experiential exercises. During day 5, the MA also receives detailed instruction on how to complete intervention fidelity forms. Monthly booster sessions are delivered in teleconferences, in which the MA is encouraged to discuss their successes, and to problem-solve around challenges.

#### Training and certifications

All research staff are trained and certified in interviewing, questionnaire administration, recruitment procedures, consenting, database use, Collaborative IRB Training Initiative (CITI) Protection of Human Subjects, and Society of Behavioral Medicine (SBM) Good Clinical Practice certification. Research staff also receive training in standardized vital sign monitoring and HIPAA compliance.

### Intervention fidelity

Intervention fidelity forms are used to track the frequency, duration, and content of all telephone outreach to participants, including blood glucose triage and “no (EMA, blood glucose, or Wisepill) data” calls for all participants, and weekly MA Health Coach feedback calls for group 3/ Dulce Digital-Me-Telephonic participants. All fidelity data are collected in a Research Electronic Data Capture (REDCap) database and reviewed on a regular basis by supervising research staff, who provide informative feedback to the MA Health Coach and other study staff on adherence to protocols and areas for improvement. The CYCORE system actively tracks the content and date/time of delivery or receipt of outgoing core content, EMA items, and feedback messages, as well as participants’ responses to EMA items. CYCORE reports are reviewed on a regular basis to ensure that intervention content is delivered as designed; any deviations from protocol or temporary system outages are tracked in REDCap.

### Outcomes assessments

Details on assessment of primary and secondary outcomes, demographic factors and other variables are shown below in Table 5.

**Table 5:** Assessments of primary and secondary outcomes, demographic, and other factors

Domain	Description	Time of assessment				Number of items
		Screening (pre-allocation)	Baseline	Month 6	Month 12	
**Primary outcome**						
HbA1c (primary), Blood Pressure, LDL-c	Laboratory visits at the clinic		X	X	X	n/a
**Secondary outcomes**						
Patient Provider communication	Select items from the Chronic Illness Resources Survey (CIRS)		X	X	X	3
Diabetes Self Management Behaviors	Summary of Diabetes Self-Care Activities Measure					7
	Rapid Assessment of Physical Activity Questionnaire					9
	Food Behavior Checklist					10
	Sedentary Behavior Questions (adapted from the International Physical Activity Questionnaire)		X	X	X	1
**Behavioral health concerns**						
Medication Adherence	Adherence to Refills and Medications Scale (adapted for diabetes by the study team)		X	X	X	11
Alcohol Consumption	CDC Behavior Risk Factor Surveillance System Survey (2014)					2
Smoking Status	CDC Behavior Risk Factor Surveillance System Survey (2014)		X	X	X	3
Diabetes Distress	Diabetes Distress Scale-17		X	X	X	17
**Demographic and social contextual factors**						
Demographic information	Race/ethnicity, nativity, language, employment, income, education, marital status		X			6
Age of Diabetes Diagnosis	Study-adapted question		X			1
Healthcare Access, Barriers, and Recent Use	CDC Behavior Risk Factor Surveillance System Survey (2014)		X			1
Health literacy	Single Item Literacy Screener		X			1
**Process Outcomes**						
Utilization and Satisfaction with Intervention	Study-adapted measure			X	X	18
RSSM^a^	Select items from the CIRS		X	X	X	3

^a^Resources and Support for Self Management

#### Primary outcomes

HbA1c, SBP, and LDL-C were chosen as clinically relevant indicators of improved quality of care, as they are consistent with national quality guidelines and are associated with decreased incidence of micro- and macrovascular disease in T2D [72]. HbA1c is the primary outcome of these, as the key indicator of glycemic management. Laboratory and BP measurements are taken at baseline, 6, and 12 months at the clinic following an 8–12-h fast. Labs are processed by Quest Diagnostics Inc., which adheres to all guidelines set forth by the College of American Pathologists [73–75]. The primary outcome of HbA1c is assayed by Immunoturbidimetry (Integra 800, Roche). Research assistants measure BP using standardized protocols and instrumentation [76, 77]. After participants are seated for 5 min, BP is measured twice, with a 2-min break between readings; notable discrepancies are resolved with a third measurement. Safety protocols outline the action to be taken should participants exhibit critical a BP value during a research visit.

#### Secondary outcomes

Several validated, self-report measures were selected to assess the secondary outcomes of patient adherence and patient-provider communication at baseline, 6, and 12 months (see Table 5). Measures were chosen based on evidence of adequate psychometric properties and appropriateness for the population. To assess improvements in patient-provider communication, participants complete the three-item, “provider support” subscale of from the Chronic Illness Resource Survey (CIRS). The CIRS and its subscales have good psychometric properties [78] and are appropriate for use in Spanish-speaking adults [79]. For brevity, seven items from the Summary of Diabetes Self-Care Activities (SDSCA) scale were selected to evaluate adherence to core diabetes self-management behaviors, including healthful eating, physical activity, glucose monitoring, and medication intake (pills and/or insulin, as applicable) [80]. In prior research, the SDSCA has demonstrated associations with other measures of diabetes self-management, adequate test-retest reliability, and sensitivity to change and has been translated and validated in Spanish [81]. Additional measures of physical activity and diet are administered to ascertain more detailed information about change in these behavioral domains. Specifically, the nine-item Rapid Assessment of Physical Activity questionnaire (RAPA) was designed to quickly assess physical activity in older adult patients and has been linguistically and psychometrically validated in English and Spanish. A single item from the International Physical Activity Questionnaire is also administered to assess sedentary behavior [82]. The Food Behavior Checklist is a ten-item measure that is used to document behavior change by assessing patient eating behaviors over the course of an intervention and has been shown to have good reliability and validity in English and Spanish [83]. Five items from the Centers for Disease Prevention and Control’s Behavior Risk Factor Surveillance Survey (2014 version, BRFSS) [84] were used to evaluate smoking and alcohol use. Finally, given the known association between emotional well-being and adherence to diabetes self-management regimens, the 17-item Diabetes Distress Scale (DDS-17) is also administered [85]. The DDS-17 produces a total score to reflect overall emotional distress related to diabetes, as well as emotional burden, regimen distress, interpersonal distress, and physician distress subscale scores. The DDS-17 has demonstrated good reliability and validity [85] and is available in Spanish [86].

#### Demographic and other variables

Demographic and other health-related factors are assessed for sample description purposes and as potential covariates for analyses. Specifically, participants complete items regarding race/ethnicity, nativity, language, employment, income, education, marital status, and age of diabetes diagnosis at baseline. They are also asked about healthcare access (using one item from the BRFSS) [84] and health literacy (using the single item literacy screener) [87], both of which are either validated in Spanish or were translated to Spanish by four independent, bilingual research assistants using the study team’s translation protocol.

#### Cost outcomes

Data from the research assessments and EHR will inform the cost-effectiveness analysis. Patient characteristics (e.g., age and gender), time-varying risk factors (HbA1c, SBP, cholesterol, smoking status), and healthcare utilization (primary and specialty care encounters, procedure codes) and costs (estimated using internal accounting systems) will be analyzed to document the cost effectiveness of the Dulce Digital, Dulce Digital-Me-Automated, and Dulce Digital-Me-Telephonic interventions from the health system perspective.

#### Process evaluation outcomes

The RE-AIM model [88, 89] will be used to evaluate feasibility, acceptability, sustainability, and dissemination and scaling potential of the Dulce Digital-Me interventions. To examine intervention *Reach*, we will compare the number of participants enrolled to those eligible and document the proportion of patients who were offered yet declined enrollment. Demographics and other factors available via the EHR will be compared between enrolled vs. not enrolled/declined patients to document differences between these groups. *Efficacy* of the Dulce Digital-Me interventions will be demonstrated via between-group differences in clinical and patient-reported outcomes over the 12-month evaluation period. Semi-structured post-study interviews and focus groups will be conducted with relevant stakeholders to gauge *adoption* rates, facilitators, and barriers. Post-study participant focus group proceedings will be audio taped and transcribed verbatim. All qualitative data will be coded and a thematic analysis approach will be applied [90, 91] using NVivo 9 software (QSR International, Victoria, Australia). In addition, the month-6 and month-12 follow-up surveys include items developed by the study team that are acceptability and feasibility with the mHealth intervention. Example items include, “How often did you read the text messages you received about managing your diabetes?” and “How much did you like receiving these text messages?” and given response options included a lot, a little, or not at all. Topic and content coverage will be tracked using the intervention fidelity forms to document *implementation.* Finally, the *maintenance* potential of the DD-Me intervention will be explored via stakeholder interviews and CAB discussions and informed via clinical and cost-effectiveness results. Findings from this multi-method process evaluation will also be used to guide intervention revisions prior to scaling.

### Participant timeline

A summary of the expected timeline for participant involvement is shown in Table 6. Enrollment occurs during a study visit scheduled in the clinic. After providing written informed consent, the baseline assessment is performed, following which randomization occurs. Group assignments are described above. The follow-up periods at months 6 and 12 were chosen to assess immediate- and longer-term impact of the intervention, respectively.

**Table 6:** Participant timeline

	Study period						
	Enrollment	Allocation	Post-Allocation				
TIMEPOINT	-t1	0	Baseline^a^	Month 1–6		Month 6	Month 12
				2–3/week	Weekly		
**ENROLLMENT**							
Screening by EHR	x						
Telephone Screening	x						
Informed Consent			x				
Allocation		x					
**INTERVENTIONS**							
All Groups							
*Brief diabetes education session*			x				
Group 1 Dulce Digital							
*Text message delivery*				x			
Group 2-Dulce Digital-Me, Automated							
*Text message delivery*				x			
*Automated text feedback*^*b*^				x	x		
Group 3-Dulce Digital-Me, Telephonic							
*Text message delivery*				x			
*Health coach feedback calls*^*b*^					x		
**ASSESSMENTS**							
Labs, BP and Anthropometric data^c^			x			x	x
Self Report Surveys^d^			x			x	x

^a^Occurs at the Neighborhood Health Care

^b^Responses and coaching based on information collected from Wisepill, connected glucose meter and ecological momentary assessments

^c^Described in outcomes

^d^Described in Table 5

## Methods: assignment of interventions

### Randomization and blinding

The study applies a block randomization scheme with equal allocation to the three groups (1:1:1) generated by the trial statistician and administered by designated research staff. The study statistician places assignments into sealed envelopes labeled with participant ID numbers. At the conclusion of the Initial Visit, the research assistant unveils the group assignment. Due to the nature of the intervention, participants are not blinded to condition. However, outcomes assessors (i.e., individuals conducting patient-reported outcomes assessments, medical records abstraction, statistical analyses) are blinded to participants’ group assignments.

## Methods: data collection, management, and analysis

### Data collection methods

Data are collected primarily from participants, and as part of the Process Aim, from stakeholders such as the MA Health Coach, and FQHC clinicians and leadership. Participant data include laboratory, blood pressure, anthropometrics, and patient-reported outcomes from baseline and follow-up visits, and qualitative data collected through post-study focus groups. The trial does not involve collecting biological specimens for storage.

The baseline assessment is conducted as part of the in-person Initial Visit, prior to randomization, at the clinic site; follow-up visits follow similar procedures and occur at 6 and 12 months post-randomization. For all assessments, trained, bilingual research assistants measure BP, and anthropometrics (height and weight). Medication review is conducted, and blood is drawn at the clinic laboratory and submitted for processing. Participants are offered a short break and a light snack, and then research assistants administer self-report assessments (Table 5). At the completion of this visit, gift cards are provided for participant time and effort.

#### Electronic health record (EHR) abstraction

All laboratory draws conducted for study purposes result in the FQHC EHR. The Scripps Health IRB approval provides permission to audit the EHR for patient identification and outcome analysis purposes. Demographic and other (static) data are extracted from the EHR for each participant upon enrollment, and clinical and health service utilization data will be abstracted for 12 months from each patient’s unique enrollment date. The EHR query will include all Neighborhood Healthcare laboratory and ambulatory clinic sites. For participants who miss a follow-up assessment but have standard of care laboratory or anthropometric values resulted in the EHR within the qualifying assessment window, these values will be used for outcome analysis purposes to enhance data completeness.

#### Patient-reported outcome assessments

Patient-reported outcomes are assessed in the participant’s preferred language, English or Spanish at baseline, month 6, and month 12 study visits by trained bilingual, bicultural research assistants using a standardized protocol. Participants are given the option of completing self-report measures as an interview or to complete a pen and paper form. Research assistants are available throughout survey administration period to assist participants as needed and check the survey responses for completeness upon finishing.

### Data management

Separate databases are maintained to track all intervention components and processes. Data are stored in password-protected Excel spreadsheets, REDCap databases, and CYCORE. All study data are entered into a secure REDCap database, which includes web-based data entry platforms for research staff to enter screening, in-person, and telephone-assessment data, and EMR abstraction. Study personnel use secure passwords to access the database. Where possible, data fields are preprogrammed to prevent entry of out of range or implausible data, and missing data are minimized by requiring that a response is entered before transitioning to the next item. Separate databases are maintained for participant tracking, recruitment and screening, EMR abstraction, intervention fidelity, and interview/self-report data. REDCap databases are stored on servers within environments that conform to HIPAA, CITI, and NIH data security regulations and are backed up daily, with external backups stored off site and exchanged weekly.

### Data quality control procedures

#### Trial steering committee (TSC)

The Dulce Digital-Me TSC is composed of the multiple principal investigators (APT; LCG) and co-investigators (ALF; SCR; JGG). The TSC convenes on a bi-monthly basis to ensure all aspects of local organization and data quality control are met, including staff training in all procedures as noted below. The TSC oversees conduct and progress throughout the course of the study including recruitment, technology, intervention oversight, and data collection and evaluation.

#### Staff training

All research staff are trained and certified in tasks required for their roles, such as questionnaire administration, recruitment procedures, consenting, database use, CITI Protection of Human Subjects, and SBM Good Clinical Practice certification. Research staff also become Scripps contractors, which includes receiving a general volunteer training and ensuring medical clearance and HIPAA compliance.

#### Quality control checks

All databases containing study data are checked for completeness and accuracy at least weekly. Baseline, follow-up assessments, and fidelity data are manually checked for completeness and accuracy. Quality control observations are performed quarterly by the study coordinator at the assessment visits for the following tasks: informed consent, blood pressure, anthropometry, survey administration, and device distribution and training. The number of follow-up surveys completed and appropriate coding of patients (e.g., refusal, deceased) are verified and confirmed. Research staff indicate their name with each survey completed and are contacted when discrepancies, errors, or omissions of data are identified.

#### Cohort retention procedures

To maximize retention and data quality, all participants receive cohort maintenance postcards at interim study points and reminder letters, calls, and text messages prior to each assessment appointment and are contacted and rescheduled if an appointment is missed. Modest monetary compensation is provided for baseline/initial visit, month 6, and month 12, respectively for time and effort rendered to all participants following each assessment visit. All research staff are carefully trained in research protocols and interviewing methods including the process of developing rapport and maintaining a friendly but professional interaction style to ensure that participants have a positive experience with the study.

### Statistical methods

#### Primary and secondary analyses

All analytic strategies will follow published standards, including intent to treat principles [92]. Preliminary data screening and cleaning will require examination of distributions for normality, outliers, and missing data patterns at both the uni- and multi-variate level. Preliminary inferential statistical testing and effect size consultation will be used to determine if random assignment has resulted in statistical equivalence between groups. Significant covariates will be added to adjust for nonequivalence. Multi-level models using full information maximum likelihood estimation will be conducted to examine changes in the target outcomes for each Aim. Analyses of clinical outcomes (HbA1c—primary; LDL-C; SBP) and patient-reported outcomes will be conducted using multi-level modeling and the appropriate link function for a target outcome. Multi-level models will be used to accommodate possible missing data and non-normally distributed variables. Analyses will include “group” (Dulce Digital, Dulce Digital-Me-Automated, Dulce Digital-Me-Telephonic) as the between-subjects factor, “time” (assessments at baseline, month 6, and month 12, with time between visits captured in months) as the within-subject factor, and a cross-level, “group-by-time” interaction effect—the primary effect of interest in this trial. Follow-up analyses will be conducted to determine the nature of the differential change between groups, using procedures outlined by Preacher, Curran, and Bauer [93]. To determine if values for primary and secondary outcomes at follow-up differ from baseline values, two dummy-coded time variables will be created and specified as within-subject predictors of the target outcome(s). The baseline assessment will be specified as the referent to each follow-up time-point (month 6, month 12), respectively. All analyses will use an intent-to-treat approach and will be conducted in IBM SPSS Statistics 22.0 (IBM, Inc., Armonk, NY, UK) and MPLUS (Muthen & Muthen, Los Angeles, CA, USA).

### Cost-effectiveness

#### Intervention costs

Intervention costs will be estimated for the Dulce Digital and DD-Me approaches from the health system perspective. Specifically, cost to maintain the CYCORE system will be tracked (both groups), and time spent by the care team nurse (both groups) and MA (DD-Me, telephonic) supporting participants via telephone-based encounters will be measured using time-logs and will be valued at the staff’s wage plus benefits. Training costs will be similarly measured and valued for both trainers and trainees. Cost of medication adherence monitors will be included. Although glucose monitors and mobile phones/texting plans (for some participants) will be provided in the present study, we expect patients to use their own phones when this program is sustained and scaled after the funding period. Overhead and administrative costs will be included.

#### Cost-effectiveness analysis (CEA) perspective and time horizon

We will estimate the long-term cost effectiveness of the Dulce Digital, Dulce Digital-Me-Automated, and Dulce Digital-Telephonic interventions from the health system perspective, excluding research and measurement costs. Observed changes in clinical outcomes (i.e., HbA1c, LDL-C, and SBP from AIM 1) and costs associated with the interventions will be used as inputs into a diabetes simulation model, the United Kingdom Prospective Diabetes Study (UKPDS) Outcomes Model, which will then be used to evaluate changes in life expectancy, quality-adjusted life expectancy, lifetime costs, and cost-effectiveness [94]. The UKPDS Outcomes Model employs an integrated system of parametric equations to estimate the absolute risk of the first occurrence of each of seven diabetes-related complications (fatal or non-fatal myocardial infarction, other ischemic heart disease, stroke, heart failure, amputation, renal failure, and eye disease) and death based on patient characteristics (e.g., age and gender) and time-varying risk factors (HbA1c, SBP, cholesterol, smoking status). Data from the UKPDS will be used to develop the predictive equations for diabetes-related complications, mortality, and progressive time paths for the risk factors, and to assign utilities conditional on disease state. Data from a large, integrated health plan were used to develop U.S. specific costs for diabetes-related complications [95].

## Methods: monitoring

### Data monitoring

Barring identifiable problems or substantial risks that would warrant discontinuation of the trial, enrollment will continue until the target sample size of 414 consented and randomized participants is reached. We are conducting bi-yearly process evaluations to monitor treatment fidelity and completion rates of key processes including ambulatory clinic visits, MA support calls, and completion of 6- and 12-month outcomes assessments.

The study follows a Data and Safety Monitoring Plan approved by the funding agency and IRB. The Data and Safety Monitoring Plan includes oversight by a three-member external data and safety monitoring committee. The safety monitoring committee is responsible for safeguarding the interests of study participants, assessing the safety and efficacy of study procedures, reviewing the data, and monitoring the overall conduct of the study. The safety monitoring committee is required to provide recommendations about starting, continuing, and stopping the study. In addition, the safety monitoring committee is asked to make recommendations, as appropriate, about the following: The efficacy of the study intervention; benefit/risk ratio of procedures and participant burden; selection, recruitment, and retention of participants; adherence to protocol requirements; completeness, quality, and analysis of measurements; amendments to the study protocol and consent forms; participant safety, and; notification of adverse events. Safety monitoring committee meetings are held yearly and are preceded by the distribution of a report of study progress, adverse events, and other issues of note.

### Harms

The primary study-related risk to participants is the potential loss of confidentially. Our data management approach includes protections to mitigate this risk. An additional risk is increased distress that could occur as a result of the assessment of behavioral health concerns, and/or in response to the intervention. The research and medical assistants are trained to remain alert to participant distress and provide urgent (e.g., crisis support services; appropriate use of 911 services) and routine psychiatric and medical care referrals (e.g., sources for outpatient healthcare) if needed during the trial. All adverse events and other unintended effects of the research and intervention, including loss of confidentiality, are monitored and are reported to the safety monitoring committee as part of the data and safety monitoring plan. The IRB determined the behavioral/educational interventions under investigation to be low risk, and interim analyses and stopping guidelines were not planned as part of this study.

### Auditing

No outside auditing is conducted as part of the trial.

### Ethics and dissemination

#### Research ethics approval

The research protocol and the informed consent form have been reviewed and approved by the reviewing (Scripps Health) and relying (San Diego State University) IRBs, with respect to scientific content and compliance with applicable research and human subject regulations. In addition, all procedures, recruitment, assessment, and intervention materials have been reviewed. All approved documents have been submitted and approved in both English and Spanish language versions. Initial IRB approval was obtained on September 28th, 2016. All modifications after the initial approval have been or will be submitted and approved by the reviewing IRBs. The responsible IRBs receive yearly progress reports, including the total number of participants enrolled and summaries of each safety and monitoring committee report, and review and approve study protocol at least annually.

#### Protocol amendments

Any protocol modifications that impact the study conduct, and/or participant risk-benefit profile, including changes in objectives, design, sample size, participant characteristics, staff changes, or significant administrative aspects, require a formal amendment to the protocol. Such amendments are submitted for approval by the relevant IRBs prior to implementation. Minor protocol corrections and/or clarifications that do not affect study conduct or participant risk/benefit profile are viewed as administrative changes and are documented internally. There have been no protocol changes that would necessitate reporting to the funding agency (i.e., changes that would affect scope of work or fulfillment of study aims). For a summary of key protocol modifications, see Appendix E).

#### Informed consent

Initial informed consent is obtained in writing, after review of the study, informed consent form, and ample time to address all questions. The informed consent form is presented in the participant’s preferred language (English or Spanish) by trained bilingual, bicultural research personnel. Informed consent is considered an ongoing process and participants are reminded of the voluntary nature of their participation at each assessment point. The informed consent form has been approved by relevant IRBs.

#### Confidentiality

Participant confidentially is considered of utmost importance by the study investigators. Steps taken to mitigate possible loss of confidentiality include the use of participant identification numbers to label all forms and data, data entry in secure password-protected REDCap data systems, and storage of all hard copy personal health information in secured, locked file cabinets within offices that operate under strict information security guidelines. The link between participant identification numbers and identity is kept for tracking and follow-up purposes only and is stored securely and separately from other data. Only trained members of the research team who require access to perform their roles have access to participant identifiers and data collected. All members of the research team are trained to ensure confidentiality and adherence to standardized procedures. All research staff directly involved with the collection and storage of research materials complete the CITI Human Subjects tutorial and the NIH Information Security Awareness Course prior to initiating data collection. Paper copies of data collected are kept in locked cabinets within a locked office. In order to adhere to new NIH data and information security guidelines, cameras are installed in the office where participant printed files are stored and in the server room where databases are stored. All research staff submit a background check prior to being hired for work with the study.

#### Declaration of interests

The study investigators have no financial or other competing interests to declare.

#### Access to data

The study investigators will have full access to all data. De-identified data will be made available to interested trainees and outside investigators for additional analyses, upon reasonable request, following reports of primary outcomes, and with appropriate data use agreement.

#### Ancillary and post-trial care

Participants will continue to receive care as usual throughout and following the trial. There is no provision of compensation for harms due to trial participation, and given the nature of the study, harms are not expected.

#### Dissemination policy

To comply with NIH data sharing policies, the study investigators, healthcare and community research partners, and members of the community will develop policies and procedures for sharing data with researchers not affiliated with the original project. We will ensure adherence to all policies and regulations of the Department of Health and Human Service, the NIH, and the participating institutions, Scripps, University of California, San Diego, and San Diego State University, including the HIPAA Privacy Rule. We will not directly share qualitative data due to potential for compromising participant identity and related ethical concerns. Broad themes and findings of these data will be shared through publications and presentations. Quantitative written data use agreements will be developed in collaboration with all research partners. Each data use agreement will require that the data be used exclusively for research purposes, for research that entails an inherent benefit to science and society and that includes a comprehensive dissemination plan (to include community and scientific audiences), that no individuals will be identifiable in any manner, that data will be secured using appropriate computer technologies, and that data will be returned or destroyed once analyses are complete. Study findings will be broadly disseminated to the academic/research community, via journal publications and conference presentations, and to stakeholder (patient, healthcare system) communities, through mechanisms such as layperson or healthcare focused reports, fact sheets, and community presentations. Optimal approaches to dissemination in each context will be developed in collaboration with stakeholder groups.

We will determine authorship using criteria developed by the International Committee of Medical Journal Editors [96]. There is no intention to engage professional writers.

## Discussion

A recent systematic review and meta-analysis of 118 randomized trials showed that DSME/S reduced HbA1c by an average of 0.74% [97], and similar findings have been reported in prior reviews [98, 99] Despite significant evidence demonstrating the benefits of DSME/S, practical barriers (e.g., work, caregiving, transportation) limit the reach of traditional or face-to-face DSME/S programs for underserved, at-risk patients. mHealth technologies have the potential to circumvent some of these obstacles.

Recently we developed *Dulce Digital*, which included culturally tailored and health literacy-sensitive educational and supportive text messages, combined with patient monitoring and transmission of blood glucose values [34]. As a “static” intervention, all Dulce Digital participants received the same content and dosage of messages (2–3 messages daily initially, with frequency tapered over 6 months). Our process evaluation indicated that Dulce Digital was both feasible and acceptable [100]; however, patients expressed a preference for intervention content tailored to their individual self-management needs and behavioral progress (i.e., an “adaptive” intervention). The DD-Me study builds on our existing infrastructure to further tailor an adaptive texting intervention and evaluate this approach. The intervention has been enhanced with the integration of the CYCORE technology. CYCORE supports this type of adaptive or personalized intervention by providing a platform for integrating information from health monitoring devices (e.g., blood glucose monitors) and mobile phone EMA of behaviors in addition to the investigative team’s experience providing tailored and interactive feedback in real time based on individuals’ wirelessly transmitted data. Further, our experience in training non-clinical staff in health coaching will allow us to examine whether this feedback is best delivered by telephone with medical assistants or via automated messaging.

The incorporation of Dulce Digital-Me in a typical primary care clinic setting to augment *existing* provider-led care team processes will provide a valuable test of real-world effectiveness, while facilitating sustainability, scalability, and dissemination. Our focus on clinical metrics (i.e., HbA1c, LDL-c, and blood pressure) that are consistent with health plan targets will increase relevance to healthcare systems and provide further incentive to sustain or adopt the Dulce Digital-Me program. The flexibility of the Dulce Digital-Me model lends itself to adaptation for other chronic conditions (e.g., arthritis, chronic pain) and for delivery by other primary care personnel to address the health needs of other underserved populations across the nation.

## Trial status

Recruitment started in June 22, 2017. The final participant’s survey/lab follow-up window was completed on June 8, 2021. Final data collection was completed on August 14, 2021.

Protocol version 5 (January 24, 2017). Substantive amendments to the original protocol (approved September 28th, 2016) are outlined in Appendix E.

## Supplementary Information

**Additional file 1.** Appendices A–E.

## Data Availability

The de-identified datasets used and/or analyzed during the current study will be made available from the study investigators following completion of study activities, on reasonable request, and with appropriate data use agreements. Materials not included in this protocol will be made available by request to the study investigators following completion of all study activities.

## Abbreviations

CIRS: Chronic Illness Resources Survey
CITI: Collaborative Institutional Training Initiative
DD-Me: Dulce Digital-Me
EMR: Electronic Medical Records
FQHC: Federally Qualified Community Health Center
HIPAA: Health Insurance Portability and Accountability Act
IRB: Institutional Review Board
MA: Medical assistant
NIH: National Institutes of Health
NHC: Neighborhood HealthCare
PROMIS: Patient-Reported Outcomes Measurement Information System
RE-AIM: Reach, Efficacy, Adoption, Implementation, and Maintenance
RSSM: Resources and Support for Self-Management Model
SES: Socioeconomic status
REDCap: Research Electronic Data Capture
UC: Usual care
US: United States
