# Supplementary material for: Dulce Digital-Me: protocol for a randomized controlled trial of an adaptive mHealth intervention for underserved Hispanics with diabetes
Source: Trials. 2022 Jan 28;23:80. doi: 10.1186/s13063-021-05899-x (PMC8796443; doi:10.1186/s13063-021-05899-x)
Supplement: Supplementary file 1 — Additional file 1. Appendices A–E. [file 13063_2021_5899_MOESM1_ESM.docx]

**Appendices**

**Appendix A.** Outline of Education Session

**Appendix B.** Provider/Electronic Health Record Report

**Appendix C.** Outgoing Text Message Frequency, by Message Type and Intervention Group

**Appendix D.** Medical Assistant Health Coach Report

**Appendix E.** Summary of Key IRB Protocol Modifications

**Appendix A.** Outline of Education Session

1. Review of diabetes
   1. Risk factors
   2. Physiology
2. Blood glucose monitoring
   1. Why monitor?
   2. Overview of hypo-, normo-, hyperglycemic ranges
3. The “ABC’s” of diabetes care
   1. A1c, blood pressure, and cholesterol goals
   2. Preventing complications
4. Nutrition
   1. My Plate guidelines
   2. Carbohydrates, salt, fats
   3. Portion sizes
5. Exercise
   1. Recommendations
   2. Safety
6. Medications
   1. Education
   2. Recommendations
7. Well-Being
   1. What is stress?
   2. Coping with stress
8. Device Training
   1. Pill box
   2. Blood glucose meter
   3. Text messages
   4. Question and Answer

**Appendix B.** Provider/Electronic Health Record Report

To facilitate patient-provider communication and accommodate physician stakeholder requests, primary care physicians are provided with a summary of blood glucose values and behavioral data from the CYCORE system prior to clinic visits.


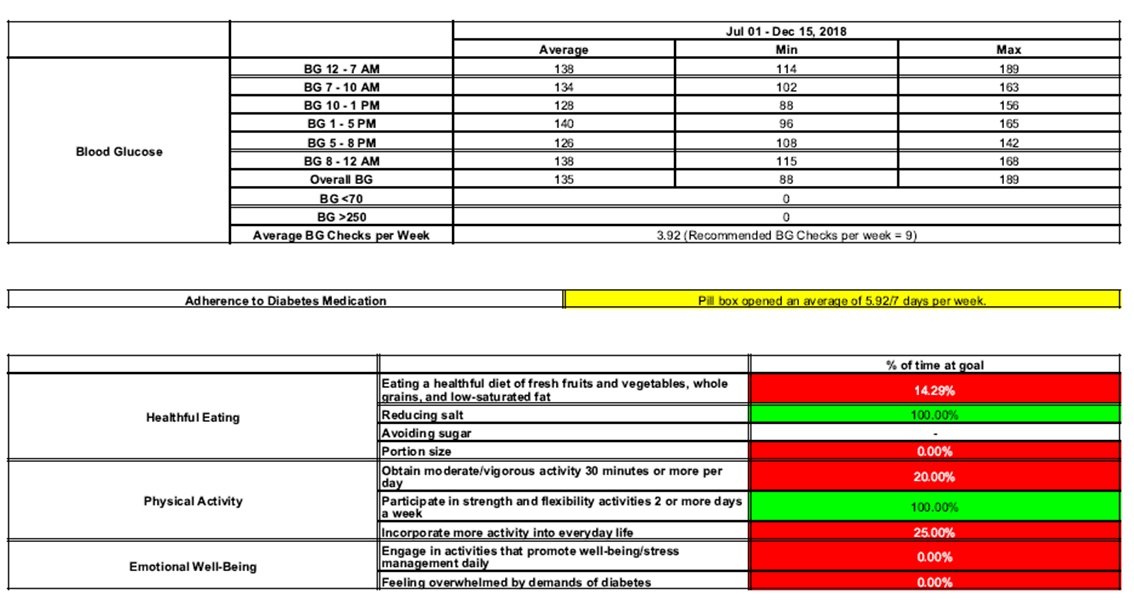


| **Appendix C.** Example Text Messages by Domain and Type | |
| --- | --- |
| **Core Content Messages** | |
| **Domain (Behavior Change Group)** | **Text Message** |
| Clinical Indicators (Goals and Planning) | Next time you check your blood sugar, ask yourself: what led to this number and what action can I take? |
| Medication (Social Support) | Does anyone else in your family take medications? Help to remind each other of the importance of taking your medications! |
| Nutrition (Antecedents) | Food cravings only last for about ten minutes. What else can you do in that time? Call a friend? Go for a walk? |
| Physical Activity (Shaping Knowledge) | Vigorous physical activity is the best for your health! Walk or jog fast enough that you breathe hard - you should be able to talk, but not sing! |
| Well-being (Regulation) | Feeling stressed? Stop and take 10 deep breaths. |
| **EMA Items & Feedback Messages** | |
| **EMA Prompt** | **Text Message Feedback Set** |
| On how many of the last 7 days have you followed your healthful eating plan? (Respond 0 to 7) | *If 5-7 days:* Wonderful! Keep up the good work!  *If 3-4 days:* You're making a really good effort! Keep trying to work towards the goal of eating healthy every day!  *If 1-2 days:* That's a good start. Remember that eating healthy every day is the goal. What can you do differently to eat healthier?  *If 0 days:* It can be hard to change eating habits at first. What goal can you set this week to get started with healthier eating? |
| On how many of the last 7 days did you do at least 30 minutes of moderate to vigorous physical activity, like brisk walking, jogging, or dancing? (Respond 0 to 7) | *If 5-7 days:* That's fantastic! Keep up the good work. Physical activity is a key to better health!  *If 3-4 days:* Great! You're very close to the goal. Try to add 1-2 more days of activity next week.  *If 1-2 days:* That's a good start. For best health, try to be active 5-7 days each week. What other exercise can you add?  *If 0 days:* It's hard to get started with physical activity. . . Can you start by walking at least 10 min each day? |
| Have you been sitting for an hour or more? (Respond Yes or No) | *If Y:* Time to take a break from sitting, it will help your health! You can stretch or just walk around for a few minutes.  *If N:* Excellent! Keep up the good work! |
| Have you done a well-being activity today? (Respond Yes or No) | *If Y:* Great! Keep it up!  *If N:* What's getting in the way? Can you find time today for a short stress relieving activity? |
| **BG Feedback Messages** | |
| **Logic Based on BG Data** | **Text Message** |
| *If >75% of BG in last 7 days 80-180* | Almost all of your blood sugars were in the 80-180 goal range this week. Keep up the good work! |
| *If 50-75% of BG in last 7 days 80-180* | Over half of your blood sugars were in the 80-180 goal range this week - Nice job! What helped to keep them low? |
| *If 25-49% of BG in last 7 days 80-180* | Less than half of your sugars were in range this week. Make sure you are taking your medications every day as prescribed. |
| *If <25% of BG in last 7 days 80-180* | Many of your blood sugars were not in the 80-180 range this week. Diabetes can be difficult! Think of what you can change to reach your goal. |
| **Medication Adherence Feedback Messages** | |
| **Logic Based on Wisepill Data** | **Text Message** |
| *If Wisepill opened 7 days/wk* | Congratulations! You took your pills every day this week! Keep up the great work! |
| *If Wisepill opened 5-6 days/wk* | Good work, you took your pills most days this week. What can you change next week to be sure you take your pills every day as prescribed? |
| *If Wisepill opened 3-4 days/wk* | We noticed that you took your pills less than 5 days this week. Be sure to place your pills in your pill box and take them exactly as prescribed. |
| *If Wisepill opened 1-2 days/wk* | You took your pills less than 3 days this week. Many things can get in the way; what can you change to remember to take your pills every day? |

**Appendix D.** Medical Assistant Health Coach Report.

Each column depicts statistics for a week (1-24) of data from device readings (blood glucose and medication adherence) and EMA responses (classified under healthful eating, physical activity, and well-being behaviors). Data is color coded based on predefined thresholds for each data type, assisting the Health Coach to quickly identify problematic values.


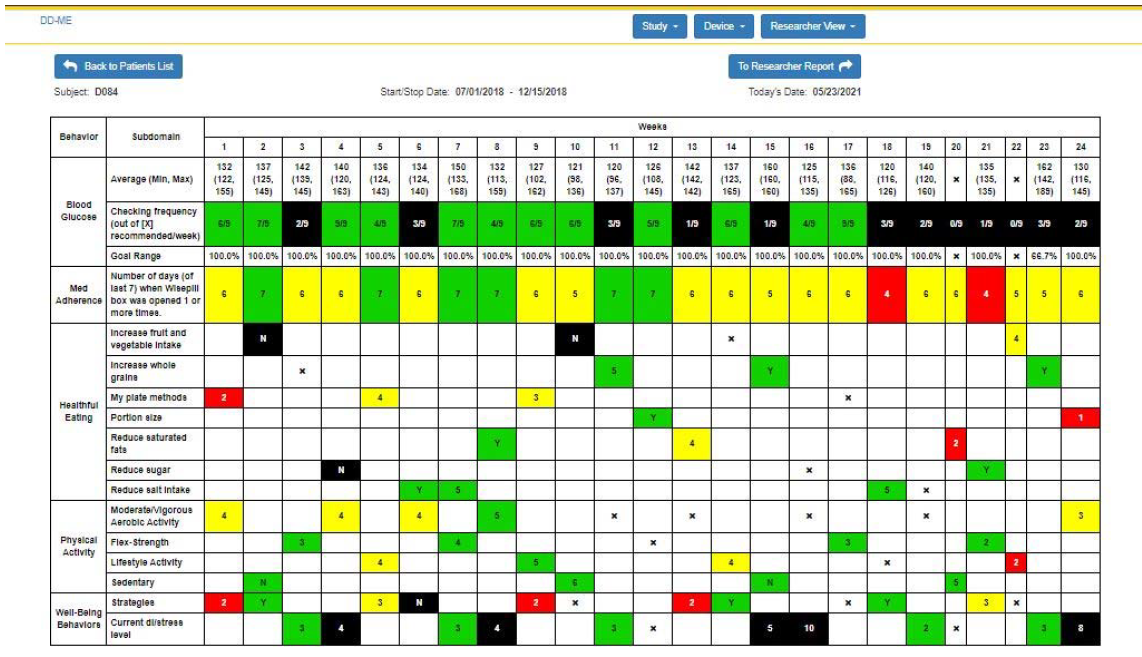


**Appendix E.** Key IRB Protocol Modifications

| **Summary of Key IRB Modifications for DD-Me** | |
| --- | --- |
| *- Initial IRB Approval for DD-Me was provided on 9/28/2016* | |
| *- Initial IRB Application included the grant application, informed consent form, protocol description (it did NOT include surveys, scripts, letters, postcards, handouts, flyers, etc.)* | |
| *- Modifications that were submitted only for adding and removing study staff (n=34) are not included in this table, Minor modifications are not included.* | |
|  | |
| **Description of Modification** | **Date Approved** |
|  |  |
| Revised Informed Consent Form to include site address, what measures will be taken, change in estimated time for follow-up assessment, clearer language for what participants will receive from the intervention | 4/5/2017 |
| Baseline and follow-up surveys, phone and in-person scripts, postcards, letters, baseline education slides and scripts, handouts, text message core content and call scripts | 4/5/2017 |
|  |  |
| Spanish Informed Consent Form and Surveys | 4/20/2017 |
|  |  |
| Protocol Changes - Removing Morisky Medication Adherence Scale and replacing it with Adherence to Refills and Medication (ARMS-D) | 6/8/2017 |
|  |  |
| *Protocol Violation Report Receipt* | 12/10/2018 |
| 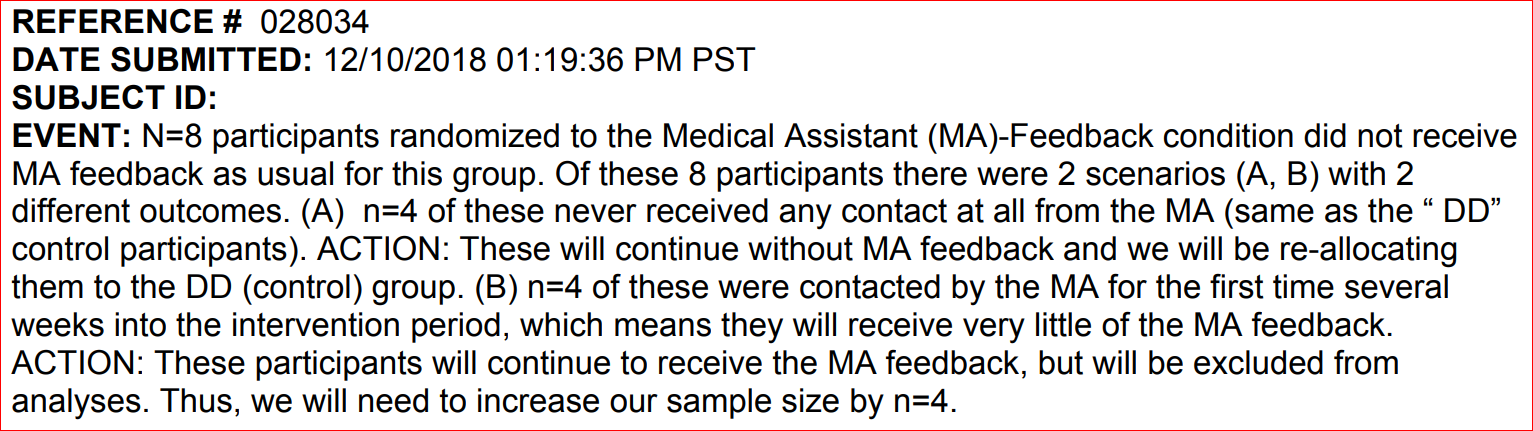   \|  \| \| --- \| |  |
|  |  |
|  |  |
|  |  |
|  |  |
| Protocol Change - Increasing planned enrollment from a target of 429 participants to a target of 433 participants, to compensate for the participants who did not receive the correct intervention (see report receipt above) | 12/18/2018 |
|  |  |
| Protocol Change - Added alternate lab visit only option for participants who did not complete the 12-Month Assessment Visit | 12/24/2019 |
|  |  |
| Protocol Changes - Modified individual telephone follow-up assessments will be performed in lieu of in-person group-based assessments due to COVID-19, and the addition of a COVID-19 check-in phone call and brief interview to provide additional support and resources will be implemented. Verbal consent will be obtained in lieu of written consent. | 5/11/2020 |
|  |  |
| Protocol Change - Addition of key informant interviews with 30 participants in lieu of planned in-person focus group interviews due to COVID-19, with key informant interview participants receiving $25 gift cards | 1/12/2021 |
|  |  |
| Protocol Change - Study Closed to Enrollment early due to COVID-19, enrollment ended officially on 3/13/2020 (NIH program officials were notified as well) | 1/20/2021 |
